# Effects of acute or short-term oral sodium bicarbonate supplementation on high-intensity intermittent exercise performance and physiological responses in team-sport athletes: a systematic review and meta-analysis

**DOI:** 10.3389/fnut.2026.1936178

**Published:** 2026-09-09

**Authors:** Xiuguo Ge, Haoyu Sun, Chuyang Wu, Shuxin Liu

**Affiliations:** 1 School of Sports and Technology, Guangzhou College of Applied Science and Technology, Zhaoqing, China; 2 Faculty of Physical Education and Sport, Yanka Kupala State University of Grodno, Grodno, Belarus

**Keywords:** exercise performance, high-intensity intermittent exercise, meta-analysis, repeated-sprint performance, sodium bicarbonate, team sports

## Abstract

**Background:**

Sodium bicarbonate (NaHCO3) ingestion can induce extracellular alkalosis, but its effects on high-intensity intermittent exercise (HIIE) performance in team sports remain unclear. This study evaluated the effects of acute or short-term NaHCO3 ingestion on HIIE performance and physiological responses in team-sport athletes.

**Methods:**

This systematic review followed the Preferred Reporting Items for Systematic Reviews and Meta-Analyses (PRISMA) 2020 statement. Six databases were searched from inception for randomized controlled trials, with the final search conducted on July 26, 2026. HIIE performance outcomes were synthesized using a covariance-adjusted three-level random-effects meta-analysis, whereas blood lactate, bicarbonate (${\text{HCO}}_{3}^{-}$), and pH were analyzed separately using two-level random-effects models. Subgroup, meta-regression, and sensitivity analyses were conducted. Risk of bias was assessed using the Cochrane Risk of Bias 2 (RoB 2) tool, and certainty of evidence was evaluated using the Grading of Recommendations Assessment, Development and Evaluation (GRADE) approach.

**Results:**

Twenty studies involving 241 athletes contributed 53 performance effect sizes. NaHCO3 ingestion had a small beneficial effect on HIIE performance (Hedges' g = 0.23, 95% CI: 0.07 to 0.39, *P* = 0.008; 95% PI: −0.22 to 0.68; I^2^ = 37.36%). No clear effects were identified for repeated-sprint performance, mean power, intermittent endurance, or work capacity, and neither subgroup analyses nor meta-regression identified significant moderating effects of sex, dose, ingestion frequency, formulation, exercise phase, or rest/work ratio. NaHCO3 ingestion increased post-exercise blood lactate (MD = 1.73 mmol/L, 95% CI: 0.97 to 2.49), ${\text{HCO}}_{3}^{-}$ (MD = 2.73 mmol/L, 95% CI: 2.16 to 3.30), and pH (MD = 0.050, 95% CI: 0.025 to 0.075). The performance findings remained stable across alternative correlation assumptions, one-primary-outcome-per-study analyses, and leave-one-study-out analyses. Certainty of evidence was low for HIIE performance and ${\text{HCO}}_{3}^{-}$ and very low for blood lactate and pH.

**Conclusion:**

Acute or short-term NaHCO3 ingestion may provide a small average improvement in HIIE performance and increase post-exercise blood lactate, ${\text{HCO}}_{3}^{-}$, and pH. However, the prediction interval crossed zero, and no consistent moderators were identified. Its practical use should therefore be individualized according to the exercise task, performance response, and gastrointestinal tolerance.

**Systematic review registration:**

https://www.crd.york.ac.uk/PROSPERO/view/CRD420261424869, identifier: CRD420261424869.

## 1 Introduction

Performance in team sports is jointly determined by technical, tactical, physical, and competitive demands. The physical demands are distinctly multidirectional, high intensity, and intermittent (1, 2). Sports such as football, basketball, and rugby require athletes to repeatedly sprint, change direction, jump, and engage in physical contests under brief and incomplete recovery and accumulating fatigue (3–5). Match analyses in football show that athletes repeatedly perform high-intensity running, which often declines as fatigue accumulates. Repeated high-intensity running is therefore considered an important indicator of the physical demands of intermittent team sports (6, 7).

High-intensity intermittent exercise (HIIE) typically consists of repeated high-intensity bouts interspersed with brief recovery periods. Performance depends not only on the intensity of each bout, but also on bout duration, recovery structure, number of repetitions, and the rest/work ratio (8, 9). Repeated high-intensity efforts increase glycolytic energy demand and are accompanied by metabolite accumulation and disturbances in acid-base homeostasis (10). Sodium bicarbonate (NaHCO_3_), an extracellular buffer, may therefore influence HIIE performance by modifying acid-base balance. Oral NaHCO_3_ generally increases blood ${\text{HCO}}_{3}^{-}$ concentration and pH, thereby increasing the transmembrane H^+^ gradient and potentially facilitating H^+^ efflux during high-intensity exercise (11, 12). However, fatigue during HIIE is also influenced by phosphocreatine resynthesis, ionic homeostasis, neuromuscular function, and energy availability (13, 14). Thus, despite its plausible physiological basis, the performance effect of NaHCO_3_ may vary with exercise mode, supplementation protocol, and individual tolerance.

Previous reviews and position statements suggest that NaHCO_3_ can enhance some high-intensity exercise tasks, particularly those lasting approximately 30 s to 12 min with substantial glycolytic involvement (12, 15). However, these findings cannot be directly extrapolated to team-sport-related HIIE. Evidence from sport-specific studies indicates that effects may depend on the sport and testing context. Gurton et al. (16) found that oral NaHCO_3_ improved repeated-sprint performance at half-time and after a simulated soccer match in male collegiate players. Conversely, Cameron et al. (17) reported that NaHCO_3_ increased blood acid-base markers but did not improve rugby-specific repeated-sprint performance in elite male players. Previous meta-analyses have also produced divergent findings. Grgic et al. (18) reported a small-to-moderate positive effect in the Yo-Yo test, whereas Lopes-Silva et al. (19) found no consistent benefit for repeated-sprint ability. Existing evidence has largely focused on single test types or individual performance outcomes. Whether NaHCO_3_ consistently improves the broader construct of team-sport-related HIIE performance therefore remains uncertain.

Existing evidence syntheses also have methodological limitations. Team-sport studies commonly report multiple related outcomes within the same trial, including repeated-sprint time, power, work capacity, intermittent-endurance performance, match-simulation performance, and phase-specific results. Ignoring within-study dependence, or retaining only one outcome, may affect the pooled estimate and its uncertainty (20, 21). We therefore used a three-level meta-analysis to retain multiple outcomes while accounting for their statistical dependence. We assessed the overall effect of acute or short-term oral NaHCO_3_ supplementation on HIIE performance in team-sport athletes. We also synthesized blood lactate, blood ${\text{HCO}}_{3}^{-}$, and pH responses to determine whether supplementation produced consistent physiological alkalosis. Subgroup analyses and exploratory meta-regression examined whether sex, dose, ingestion frequency, formulation, exercise phase, and rest/work ratio moderated the performance effect.

## 2 Methods

This systematic review and meta-analysis was reported in accordance with the Preferred Reporting Items for Systematic Reviews and Meta-Analyses (PRISMA) 2020 statement (22). The review was prospectively registered in the International Prospective Register of Systematic Reviews (PROSPERO; CRD420261424869).

### 2.1 Literature search

PubMed, Web of Science, the Cochrane Library, and Scopus were searched from inception to June 15, 2026. The search was updated on July 26, 2026, when Embase and SPORTDiscus were added. Search strategies combined controlled vocabulary and free-text terms using Boolean operators (AND and OR) and truncation. Search fields and expressions were adapted to the syntax of each database (23). Key terms included “sodium bicarbonate,” “bicarbonate loading,” “repeated sprint,” “high-intensity intermittent exercise,” “team sport,” and “exercise performance.” To maximize sensitivity, the searches were not restricted to acute or short-term supplementation. Intervention duration was assessed during full-text screening against the prespecified eligibility criteria. Reference lists of included studies and relevant reviews were also screened. Complete database-specific search strategies are provided in Supplementary Table S1.

### 2.2 Inclusion and exclusion criteria

Studies were selected using prespecified population, intervention, comparator, outcome, and study-design (PICOS) criteria. Studies were eligible when: (1) participants were healthy team-sport athletes, defined by the original authors as athletes engaged in organized team-sport training or competition, including football, basketball, rugby, field hockey, or water polo, with no restrictions on age, sex, or competitive level; (2) the intervention involved acute or short-term oral NaHCO_3_ supplementation, with acute supplementation defined as a single or divided dose within 24 h before testing and short-term supplementation as continuous or intermittent ingestion for no more than 7 days; (3) the comparator was a bicarbonate-free placebo, such as sodium chloride, cellulose, maltodextrin, or cornflour, administered under conditions matching the intervention; (4) at least one team-sport-related HIIE performance outcome was reported, including sprint time, distance completed, power, total work, or sport-specific skill performance; and (5) the study used a randomized parallel, crossover, or repeated-measures controlled design and reported sufficient data to calculate an effect size. Team-sport-related HIIE was defined as a repeated-sprint, intermittent-endurance, or team-sport simulation test comprising at least two high-intensity exercise bouts separated by incomplete recovery. Studies were excluded when: (1) participants were unhealthy, were not team-sport athletes, or were animals; (2) the intervention was not oral NaHCO_3_ or no corresponding placebo was used; (3) the exercise task or outcome did not meet the prespecified definition, or data were insufficient to calculate an effect size; or (4) the report was a review, conference abstract, protocol, thesis, or other non-peer-reviewed publication. For multi-arm or multi-condition studies, only independently isolatable comparisons between NaHCO_3_ and placebo were extracted. Environmental, pre-fatigue, or match-simulation conditions were required to be identical between treatments.

### 2.3 Study selection

Records were imported into EndNote 21 and deduplicated using automated identification followed by manual verification. Two reviewers independently screened titles and abstracts and then assessed full texts against the prespecified eligibility criteria. Disagreements were resolved through discussion and, when necessary, consultation with a third reviewer. The selection process was documented using a PRISMA 2020 flow diagram.

### 2.4 Data extraction and transformation

Two reviewers independently extracted and cross-checked the data. Extracted information included study design, participant characteristics, intervention and comparator protocols, exercise tests, outcomes, and gastrointestinal adverse events. For crossover trials, treatment sequence, washout period, period effects, and carryover effects were additionally extracted. The primary outcome was team-sport-related HIIE performance. Secondary outcomes were blood lactate, ${\text{HCO}}_{3}^{-}$, and pH.

The primary analysis included all eligible performance outcomes for which an effect size could be calculated. When outcomes were derived repeatedly from the same data, overlapped, or represented alternative expressions of the same outcome, only one was retained. For the sensitivity analysis retaining one effect per study, the primary outcome specified by the original study was prioritized. When no primary outcome was specified, the hierarchy was overall or mean performance, distance completed or time to exhaustion, and total work or mean power. Phase-specific, peak, or performance-decrement outcomes were selected only when these measures were unavailable. When a study included multiple independent samples, a primary outcome was selected for each sample before the effects were combined at the study level. Outcome-level extraction data are provided in Supplementary Table S2.

For quantitative synthesis, sample size, mean, and dispersion were extracted for the NaHCO_3_ and placebo (PLA) conditions. When a standard error (SE) was reported, it was converted to a standard deviation (SD) using the method recommended in the Cochrane Handbook (24):


$$\begin{array}{l} SD\;=\;SE\;\times \;\sqrt{n} \end{array}$$

When only confidence intervals, paired differences, change scores, or adjusted estimates were reported, effect sizes and sampling variances were calculated from the sample size, degrees of freedom, and relevant statistics. Data presented only in figures were extracted using WebPlotDigitizer (25).

Because performance outcomes were reported in different units, effect sizes were expressed as small-sample-corrected Hedges' g (26):


$$\begin{array}{l} g\;=\;\frac{\Gamma \left(\frac{m}{2}\right)}{\sqrt{\frac{m}{2}}\Gamma \left[\frac{m-1}{2}\right]}\;\times \;\frac{{\bar{X}}_{NaHCO_{3}}-{\bar{X}}_{PLA}}{S} \end{array}$$

In this equation, S is the standardizer, m is the degrees of freedom, and Γ is the gamma function. For parallel-group trials, the pooled within-group SD was used. For crossover trials, the square root of the mean of the two condition variances was used as the standardizer. None of the 18 crossover or repeated-measures studies reported the paired correlation between conditions. Following the correlation-imputation principle in the Cochrane Handbook, r was set to 0.50 in the primary analysis (24). Effect sizes for outcomes in which lower values indicated better performance were multiplied by −1, so that positive values consistently favored NaHCO_3_. For blood lactate, ${\text{HCO}}_{3}^{-}$, and pH, the first available measurement after the final exercise bout was extracted. Additional data transformations and equations for the standardizer, degrees of freedom, and sampling variance for each study design are provided in the Supplementary methods.

### 2.5 Risk of bias and certainty of evidence

Risk of bias was assessed using the revised Cochrane risk-of-bias tool for randomized trials (RoB 2) (27). Parallel-group trials were assessed with the standard tool and crossover trials with the crossover-specific version. Assessments targeted the effect of assignment to intervention and were conducted for outcomes included in the quantitative synthesis. Period and carryover effects were additionally assessed for crossover trials. Signaling-question responses and outcome-specific justifications were recorded before domain judgments were made. Outcomes with identical domain-level judgments within a study were combined for graphical presentation, whereas discordant outcomes were displayed separately. Overall risk of bias was classified as low risk, some concerns, or high risk. Certainty of evidence was assessed using the Grading of Recommendations Assessment, Development and Evaluation (GRADE) approach (28) for team-sport-related HIIE performance and the main physiological outcomes. Evidence was rated across 5 domains and classified into 4 levels: high, moderate, low, or very low. The domains were risk of bias, inconsistency, indirectness, imprecision, and publication bias. Two reviewers independently completed all assessments. Disagreements were resolved by discussion and, when necessary, consultation with a third reviewer.

### 2.6 Statistical analysis

All analyses were performed in R (version 4.5.0), primarily using the metafor (version 4.8-0), clubSandwich (version 0.7.0), and ggplot2 (version 4.0.2) packages (29). Because individual studies could report multiple eligible performance outcomes, we fitted a three-level random-effects meta-analysis with effect sizes nested within studies (20, 21):


$$\begin{array}{l} y_{ij}\;=\;\mu \;+\;u_{j}+v_{ij}+e_{ij} \end{array}$$

Here, y_ij_ is effect size i from study j, μ is the overall mean effect, u_j_ is the between-study random effect, v_ij_ is the within-study random effect, and e_ij_ is the sampling error. The model decomposed total variability into sampling error, within-study heterogeneity, and between-study heterogeneity. Because sampling errors for multiple effect sizes derived from the same analytic sample may be correlated, an approximate within-study sampling variance-covariance matrix was constructed. The covariance between two effect sizes was calculated as follows:


$$\begin{array}{l} Cov\left(y_{i},y_{j}\right)=\;\rho \sqrt{v_{i}v_{j}} \end{array}$$

Here, v_i_ and v_j_ are the sampling variances of the two effect sizes, and ρ is the assumed sampling correlation between effect sizes from the same analytic sample. Covariances between independent samples were set to 0, and ρ was set to 0.50 in the primary analysis. Model parameters were estimated using restricted maximum likelihood (REML). Inference for the overall effect used cluster-robust variance estimation with the CR2 small-sample adjustment, with studies as clusters and Satterthwaite-adjusted degrees of freedom (df). This approach was used to calculate the standard error, 95% confidence interval (CI), and two-sided *P* value (30, 31). The 95% prediction interval (PI) was calculated as follows (32):


$$\begin{array}{l} \hat{\mu }\pm t_{0.975,\text{df}}\sqrt{{{\text{S}{\text{E}}_{\text{CR}2}}^{2}+{\tau_{level2}}^{2}+\tau_{level3}}^{2}} \end{array}$$

The CR2-adjusted standard error represents uncertainty in the overall mean effect, whereas the within-study and between-study variance components were incorporated into the prediction interval. Pooled performance effects were interpreted using Hedges' g: g <0.20 was considered trivial, 0.20 ≤ g <0.50 small, 0.50 ≤ g <0.80 moderate, and g ≥ 0.80 large (33). Blood lactate, ${\text{HCO}}_{3}^{-}$, and pH were analyzed as raw-scale mean differences (MDs) using separate two-level random-effects meta-analyses. Independent analytic samples from the same publication were entered separately. Model parameters were estimated using REML with Knapp-Hartung small-sample adjustment. Heterogeneity was assessed using Q, τ^2^, and I^2^. In the three-level model, I^2^ was further partitioned into within-study and between-study components. I^2^ values of <25%, 25% to <50%, 50%−75%, and >75% were interpreted as low, moderate, substantial, and high heterogeneity, respectively. Interpretation also considered effect direction, confidence intervals, and the number of studies (24).

To compare pooled effects across performance domains, we conducted prespecified exploratory domain analyses. Repeated-sprint time, mean power, intermittent endurance, and work capacity were meta-analyzed when at least 3 studies were available. Each domain was analyzed using the same three-level model as the primary analysis. Gastrointestinal adverse events were not quantitatively pooled because assessment tools and reporting times differed substantially. Instead, dose, formulation, ingestion timing, symptom type, and severity were summarized qualitatively by study.

Subgroup analyses and meta-regression were conducted to explore heterogeneity and potential moderators. Each subgroup required at least 3 studies, and meta-regression of a continuous variable required at least 10 independent studies. Subgroup variables were sex (male or female), dose (0.3 or 0.4 g·kg^−1^), ingestion frequency (single or ≥2 ingestions), formulation (solid or liquid), and exercise phase (early, middle, or late). The number of studies, number of effect sizes, and *P* value for the subgroup difference were reported. Analyses with few studies or markedly imbalanced groups were interpreted as exploratory. The rest/work ratio was calculated as recovery duration divided by the duration of the adjacent high-intensity bout and entered as a continuous moderator in a univariable linear meta-regression. A *post hoc* exploratory quadratic model was fitted by adding a squared term. Given the limited number of studies, the linear analysis was considered exploratory and the quadratic model hypothesis-generating.

Several sensitivity analyses assessed the robustness of the primary findings. (1) To examine the assumed paired correlation, r was set to 0.30, 0.70, and 0.90, and effect sizes and sampling variances were recalculated for crossover trials. (2) Because the true sampling correlation between effect sizes from the same analytic sample was unknown, ρ values of 0.30, 0.70, and 0.90 were used to reconstruct the sampling variance-covariance matrix and refit the three-level model. (3) One primary effect was retained per study according to the prespecified outcome hierarchy. For studies with multiple independent samples, the primary outcome was selected separately for each sample and then combined into a study-level effect using inverse-variance weighting. This dataset was reanalyzed using a REML random-effects model with Knapp-Hartung adjustment (34). (4) To reduce potential confounding by training status and exercise protocol, analyses were restricted separately to trained team-sport athletes and studies using team-sport-specific HIIE protocols. (5) Leave-one-study-out analyses sequentially removed each study and all its associated effect sizes. The primary model was refitted, and changes in the pooled effect, 95% confidence interval, *P* value, and heterogeneity were examined.

When at least 10 studies were available, small-study effects were assessed at both the effect-size and study levels. At the effect-size level, contour-enhanced funnel plots and three-level Egger regression with study-clustered CR2 adjustment were used (35–37). At the study level, one prespecified primary effect was retained per study. Effects from multiple independent samples were first combined, after which a funnel plot and conventional Egger test were produced. The study-level analysis was the primary assessment, and the effect-size-level analysis was exploratory. Given the limited number of studies, a non-significant result was not interpreted as evidence that publication bias was absent.

## 3 Results

### 3.1 Study selection

**Figure 1:**
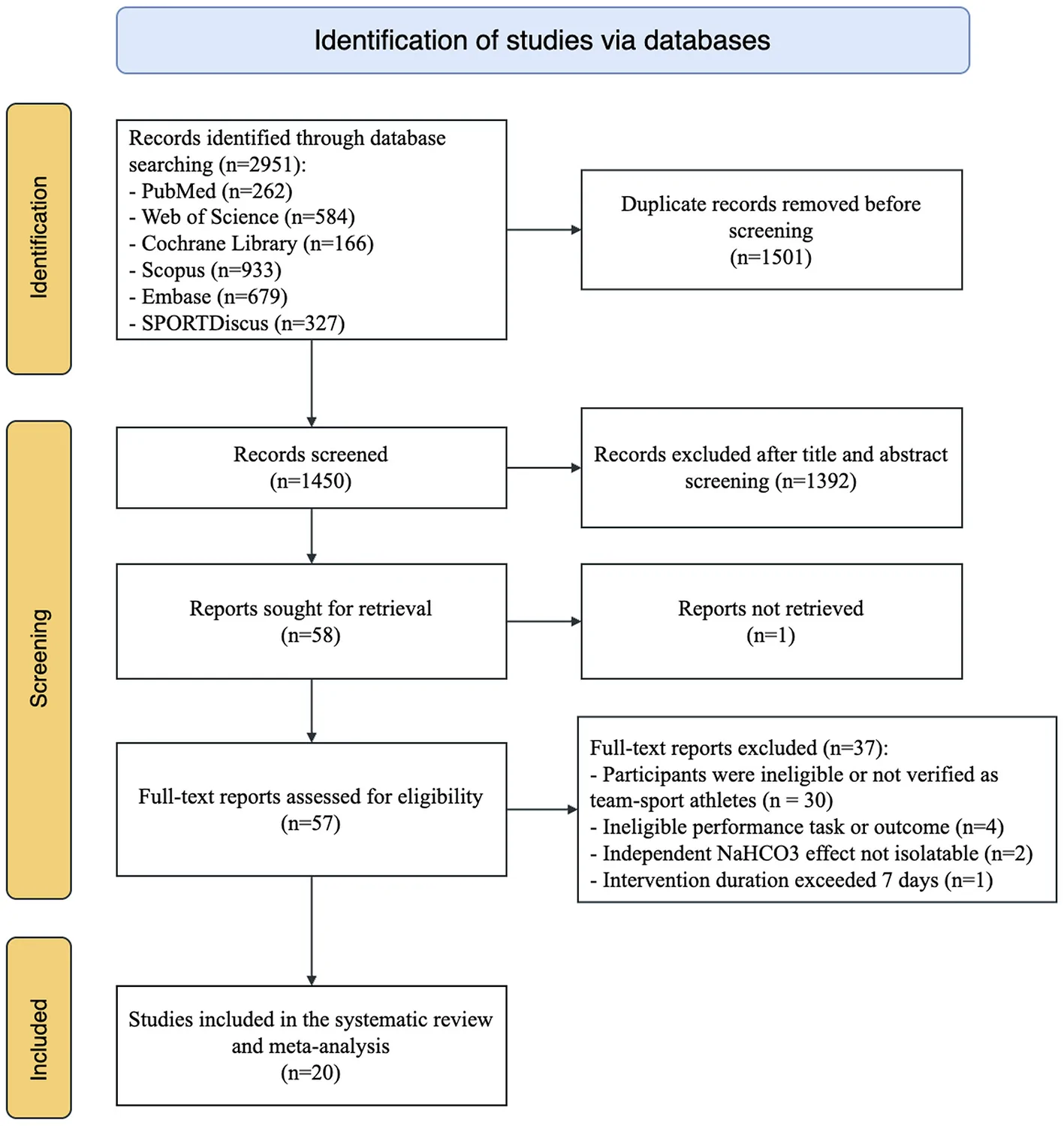
PRISMA flow diagram of study selection.

The database searches identified 2,951 records from PubMed, Web of Science, the Cochrane Library, Scopus, Embase, and SPORTDiscus. After removing 1,501 duplicates, 1,450 records were screened by title and abstract, and 1,392 were excluded. We sought 58 full-text reports, of which 1 could not be retrieved, leaving 57 reports for eligibility assessment. A total of 37 reports were excluded because the population or sport was ineligible or team-sport-athlete status could not be verified (*n* = 30), the exercise test or outcome was ineligible (*n* = 4), the independent effect of oral NaHCO_3_ could not be isolated (*n* = 2), or the intervention exceeded 7 days (*n* = 1). Overall, 20 studies were included in the systematic review and quantitative synthesis. The selection process is shown in Figure 1, and excluded full-text reports and reasons are listed in Supplementary Table S3.

### 3.2 Characteristics of included studies

A total of 20 studies published between 2004 and 2026 were included (Table 1) (10, 16, 17, 38–54), comprising 241 team-sport athletes. Individual sample sizes ranged from 7 to 25. The included sports were football (7 studies) (16, 38, 40, 41, 43, 45, 50), basketball (3 studies) (42, 47, 51), field hockey (2 studies) (44, 46), water polo (1 study) (53), and rugby (1 study) (17). The remaining 6 studies included mixed team-sport athletes or did not further specify the sport (10, 39, 48, 49, 52, 54). Participants spanned different ages and competitive levels, including youth, recreational, collegiate, professional, and elite athletes. Mean age ranged from 15.0 to 26.5 years. A total of 14 studies included only male participants (*n* = 178), and 6 included only female participants (*n* = 63). A total of 18 studies used crossover or repeated-measures designs, and 2 used parallel-group designs (40, 52). NaHCO_3_ doses ranged from 0.2 to 0.5 g·kg^−1^, with 13 studies using 0.3 g·kg^−1^. Gurton et al. (40) and Delextrat et al. (47) used short-term repeated supplementation, whereas the remaining studies primarily used single or divided acute doses before exercise. Tests included repeated sprints, HIIE protocols, and team-sport simulations. Outcomes included sprint performance, distance completed, power output, sport-specific skills, and blood biomarkers. Among the 18 crossover or repeated-measures studies, 14 used randomized or counterbalanced treatment sequences and 15 reported washout periods of 4–14 days. Reporting of period and carryover effects was generally limited. Only 1 study explicitly tested carryover and found no significant effect (43). Treatment sequence, washout, period effects, and carryover effects are detailed in Supplementary Table S4.

**Table 1:** Characteristics of the included studies.

References	Study design	Participants/sport and age	Analyzed sample	Supplementation protocol	Washout period	Exercise test/protocol	Outcomes
Barata et al. (38)	Randomized, double-blind, placebo-controlled crossover trial	Recreational female football players; 20 ± 2 y	*n* = 11	NaHCO_3_: 0.2 g·kg^−1^ at −2 h + 0.1 g·kg^−1^ at −1 h (capsules); PLA: cellulose	≥1 week	Cycle-ergometer RSA: 3 sets of 6 × 6-s maximal sprints with 24-s active recovery and 5-min recovery between sets	Mean power; blood lactate
Gurton et al. (40)	Randomized, double-blind, placebo-controlled parallel trial	Male collegiate soccer players; age NR	*n* = 16 (NaHCO_3_: 8; PLA: 8)	NaHCO_3_: 0.3 g·kg^−1^ after muscle-damaging exercise and at −2 h on Day 3 (capsules); PLA: cornflour	NA (parallel)	Soccer-specific performance battery performed after exercise-induced muscle damage	Yo-Yo IR2 distance; agility time; repeated-sprint time; ${\text{HCO}}_{3}^{-}$; pH
Gurton et al. (16)	Block-randomized, double-blind crossover trial	Collegiate male soccer players; 24 ± 3 y	*n* = 10	NaHCO_3_: 3 × 0.1 g·kg^−1^ over 30 min (capsules); PLA: matched capsules	5–7 days	SAFT90 soccer simulation with an 8 × 25-m repeated-sprint test before, at half-time, and after SAFT90	Fastest and mean sprint times; blood lactate; ${\text{HCO}}_{3}^{-}$; pH
Gurton et al. (39)	Block-randomized, double-blind crossover trial	Recreationally trained male team-sport athletes; 26.5 ± 5.8 y	*n* = 14	NaHCO_3_: 0.3 g·kg^−1^ in three doses over 30 min (capsules); PLA: matched capsules	5–7 days	Team-sport battery: 8 × 25-m repeated sprints followed by Yo-Yo IR2	Sprint times; Yo-Yo IR2 distance; blood lactate
Dahmer et al. (41)	Randomized repeated-measures trial	Male football players; 19.2 ± 0.7 y	*n* = 14	NaHCO_3_: 0.3 g·kg^−1^ at −40 min (juice); PLA: juice	NR	Yo-Yo intermittent recovery test with repeated 2 × 20-m shuttle runs and 10-s active recovery	Yo-Yo distance
Ansdell and Dekerle (42)	Randomized, double-blind crossover trial	Active male basketball players; 21 ± 1 y	*n* = 10	NaHCO_3_: 2 × 0.2 g·kg^−1^ at −90 and −60 min (solution); PLA: NaCl solution	7 days	Basketball match simulation comprising four modified LIST quarters with 15-m sprints and layups	15-m sprint time
Guimarães et al. (43)	Randomized, double-blind crossover trial	Semiprofessional adolescent male soccer players; 15 ± 1 y	*n* = 15	NaHCO_3_: 0.3 g·kg^−1^ at −90 min (solution); PLA: NaCl	7 days	RAST: 6 × 35-m maximal running sprints with 10-s passive recovery	Total sprint time; mean and peak power; blood lactate; ${\text{HCO}}_{3}^{-}$; pH
Durkalec-Michalski et al. (44)	Randomized, double-blind, placebo-controlled crossover trial	Trained male field-hockey players; Acute cohort: 22.2 ± 2.6 y	*n* = 12 (acute cohort)	NaHCO_3_: 0.2 g·kg^−1^ at −2 h (tablets); PLA: matched tablets	14 days	Hockey-specific intermittent field test plus two 30-s Wingate tests	Hockey-test time; Wingate mean and peak power; blood lactate; ${\text{HCO}}_{3}^{-}$; pH
Jones et al. (45)	Randomized, double-blind, placebo-controlled crossover trial	Professional and amateur male soccer players; Professional: 21.7 ± 2.1; amateur: 22.8 ± 1.2 y	*n* = 14 (professional: 7; amateur: 7)	NaHCO_3_: 0.3 g·kg^−1^ at approximately −90 to −100 min (capsules); PLA: microcrystalline cellulose	NR	5 × 6-s maximal cycle sprints with 24-s recovery	Mean sprint power; blood lactate
Macutkiewicz and Sunderland (46)	Randomized placebo-controlled crossover trial	Elite female field-hockey players; 23 ± 5 y	*n* = 8	NaHCO_3_: 0.3 g·kg^−1^, split dose (capsules); PLA: maltodextrin	≥5 days	Four LIST sets interspersed with field-hockey skill tests	LIST sprint time; field-hockey skill; blood lactate; ${\text{HCO}}_{3}^{-}$; pH
Delextrat et al. (47)	Double-blind, placebo-controlled crossover trial	Female university basketball players; 23.3 ± 3.4 y	*n* = 15	NaHCO_3_: 0.4 g·kg^−1^·day^−1^ for 3 days in three daily doses (capsules); PLA: calcium carbonate	7 days	Modified Basketball Exercise Simulation Test with 17 sprint, jump, and shuffle circuits	Total sprint and circuit time; jump height; performance decrement; blood lactate
Mündel et al. (48)	Randomized, double-blind crossover trial	Competitive male team-sport athletes; age NR	*n* = 10	NaHCO_3_: 0.5 g·kg^−1^ in three doses, 4 h apart (beverage); PLA: 0.2 g·kg^−1^ NaCl	7 days	Two 30-s Wingate tests with 5-min recovery in the heat	Second-test total work and peak power; blood lactate; ${\text{HCO}}_{3}^{-}$; pH
Marriott et al. (49)	Randomized crossover trial	Sub-elite male team-sport athletes; 20.8 ± 1.4 y	*n* = 12	NaHCO_3_: 0.4 g·kg^−1^ over 30 min from −90 min (capsules); PLA: matched capsules	≥4 days	Seventeen minutes of intense arm-cranking pre-fatigue followed by Yo-Yo IR2	Yo-Yo IR2 distance; blood lactate
Cholewa et al. (50)	Randomized, double-blind, placebo-controlled crossover trial	Division III collegiate male soccer players; age NR	*n* = 7	NaHCO_3_: 0.3 g·kg^−1^ at −60 min; PLA: cornflour	NR	Soccer-specific Yo-Yo IR2 conditioning test	Yo-Yo IR2 distance; blood lactate; pH
Afman et al. (51)	Randomized crossover trial	Well-trained male basketball players; 21 ± 2 y	*n* = 7	NaHCO_3_: 2 × 0.2 g·kg^−1^ at −90 and −20 min (solution); PLA: NaCl solution	7 days	Validated basketball simulation comprising four LIST-based blocks with layups	15-m sprint time; blood lactate; ${\text{HCO}}_{3}^{-}$; pH
Ducker et al. (52)	Randomized, placebo-controlled four-arm parallel trial	Competitive male Australian football, field-hockey, and soccer athletes; NaHCO_3_: 21 ± 3; PLA: 19 ± 3 y	*n* = 12 (NaHCO_3_: 6; PLA: 6)	NaHCO_3_: 0.3 g·kg^−1^ at −60 min (capsules); PLA: NaCl	NA (parallel)	Repeated-sprint test: 3 sets of 6 × 20-m maximal sprints, departing every 25 s, with 4-min active recovery between sets	Total sprint time; blood lactate
Tan et al. (53)	Randomized, double-blind crossover trial	Elite female water-polo players; 23.7 ± 3.0 y	*n* = 12	NaHCO_3_: 0.3 g·kg^−1^ at −90 min (capsules); PLA: matched capsules	7 days	Fifty-nine-minute water-polo match simulation containing repeated 10-m sprint-swim bouts	Mean sprint-swim time; blood lactate; ${\text{HCO}}_{3}^{-}$; pH
Cameron et al. (17)	Randomized, double-blind crossover trial	Elite male rugby-union players; 21.6 ± 2.6 y	*n* = 25	NaHCO_3_: 0.3 g·kg^−1^ at approximately −65 min (isotonic drink); PLA: NaCl drink	7 days	Rugby-specific repeated-sprint test: 10 × 40-m sprints at 30-s intervals after rugby-specific activity	Sprint times; blood lactate; ${\text{HCO}}_{3}^{-}$; pH
Bishop and Claudius (54)	Randomized, double-blind crossover trial	Female team-sport athletes; 19 ± 1 y	*n* = 7	NaHCO_3_: 2 × 0.2 g·kg^−1^ at −90 and −20 min (capsules); PLA: NaCl	7 days	Cycling intermittent-sprint test comprising two 36-min halves with repeated all-out sprints	Total work; mean peak power; ${\text{HCO}}_{3}^{-}$
Bishop et al. (10)	Randomized, counterbalanced crossover trial	Recreational female team-sport players; 19 ± 1 y	*n* = 10	NaHCO_3_: 0.3 g·kg^−1^ at −90 min; PLA: NaCl	7 days	5 × 6-s cycle sprints departing every 30 s	Total work; sprint work; peak power; blood lactate; ${\text{HCO}}_{3}^{-}$; pH

For multi-arm or multi-condition studies, only comparisons between oral NaHCO_3_ alone and the corresponding PLA were included. ${\text{HCO}}_{3}^{-}$, bicarbonate; LIST, Loughborough Intermittent Shuttle Test; NA, not applicable; NaCl, sodium chloride; NR, not reported; PLA, placebo; RAST, Running-based Anaerobic Sprint Test; RSA, repeated-sprint ability; SAFT90, Soccer Aerobic Fitness Test; WAnT, Wingate Anaerobic Test; Yo-Yo IR2, Yo-Yo Intermittent Recovery Test Level 2; –, time before exercise.

### 3.3 Risk of bias assessment

**Figure 2:**
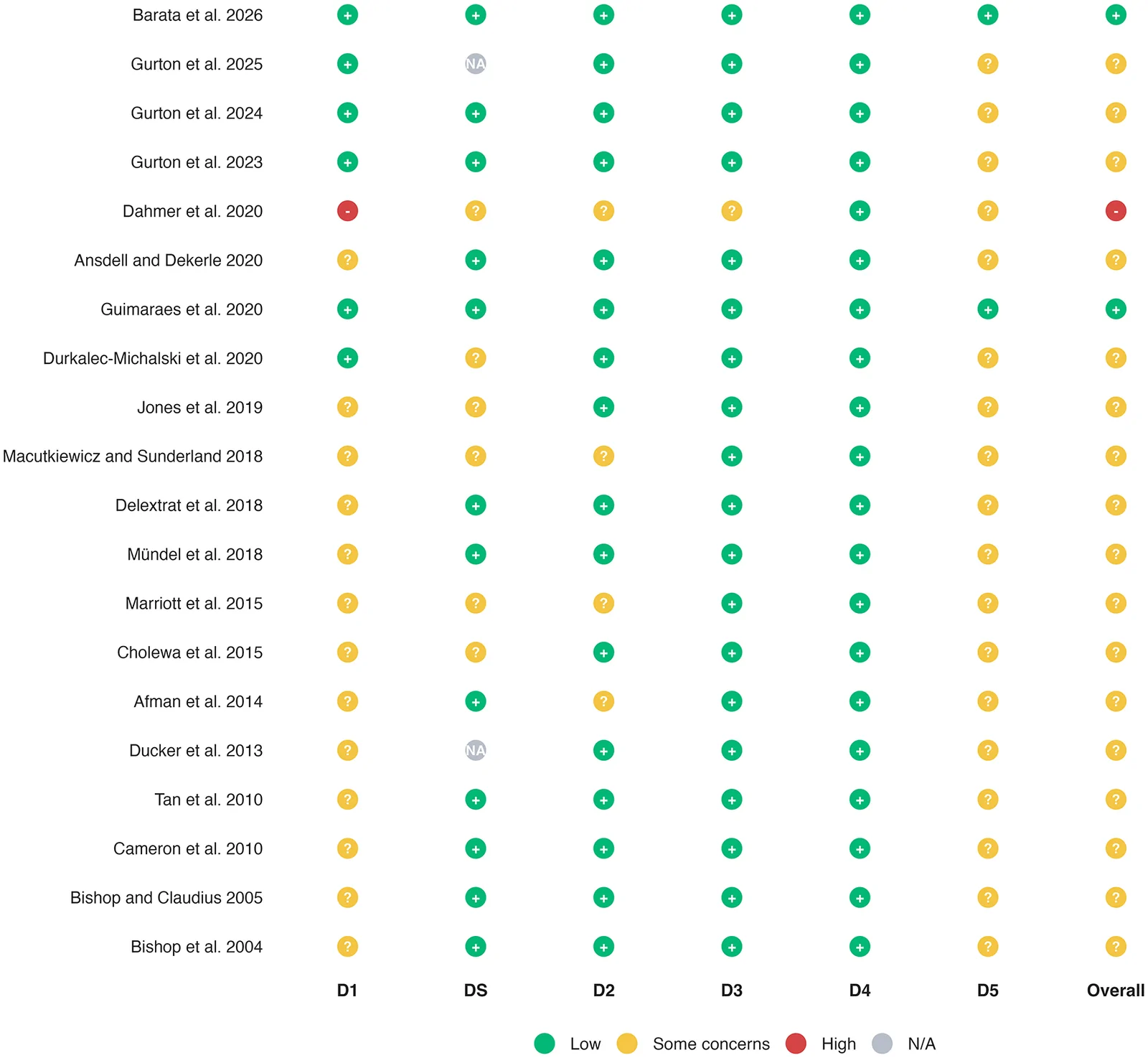
Risk of bias assessment of the included studies.

Risk of bias was assessed for the 20 included studies using Cochrane RoB 2 (Figure 2). Overall, 2 studies were judged at low risk, 17 raised some concerns, and 1 was at high risk. For the randomization process, 6 studies were at low risk and 13 raised some concerns because sequence generation or allocation concealment was insufficiently reported. Dahmer et al. (41) was judged at high risk because the randomization information was inconsistent with the reported study design. Among the 18 crossover or repeated-measures studies, 12 were at low risk for period and carryover effects. The remaining 6 raised some concerns because treatment sequence, period effects, or carryover were insufficiently reported or addressed. For deviations from intended interventions, 16 studies were at low risk and 4 raised some concerns because blinding or intervention implementation was insufficiently described. All studies except Dahmer et al. (41) were at low risk for missing outcome data, and all studies were at low risk for outcome measurement. Selective reporting was the most common concern. Only Barata et al. (38) and Guimarães et al. (43) were at low risk, whereas the other 18 studies raised some concerns because no accessible prospective analysis plan was identified.

### 3.4 GRADE assessment

The certainty of evidence was assessed using GRADE (Supplementary Table S5). Evidence for HIIE performance was rated low after downgrading once for risk of bias and once for imprecision. The total sample size was below the prespecified optimal information size, and the 95% CI (0.07 to 0.39) crossed the important-effect threshold of g = 0.20. Indirectness was not downgraded because results restricted to trained athletes or team-sport-specific HIIE protocols were consistent with the primary analysis. Evidence was rated very low for blood lactate because of risk of bias, inconsistency, and imprecision; low for ${\text{HCO}}_{3}^{-}$ because of risk of bias and imprecision; and very low for pH because of risk of bias, inconsistency, and imprecision. Sample sizes for all physiological outcomes were below the optimal information size, and the 95% prediction intervals for blood lactate and pH crossed zero. No outcome was downgraded for publication bias, although publication bias could not be excluded because few independent studies were available.

### 3.5 Meta-analysis results

#### 3.5.1 Primary analysis

**Figure 3:**
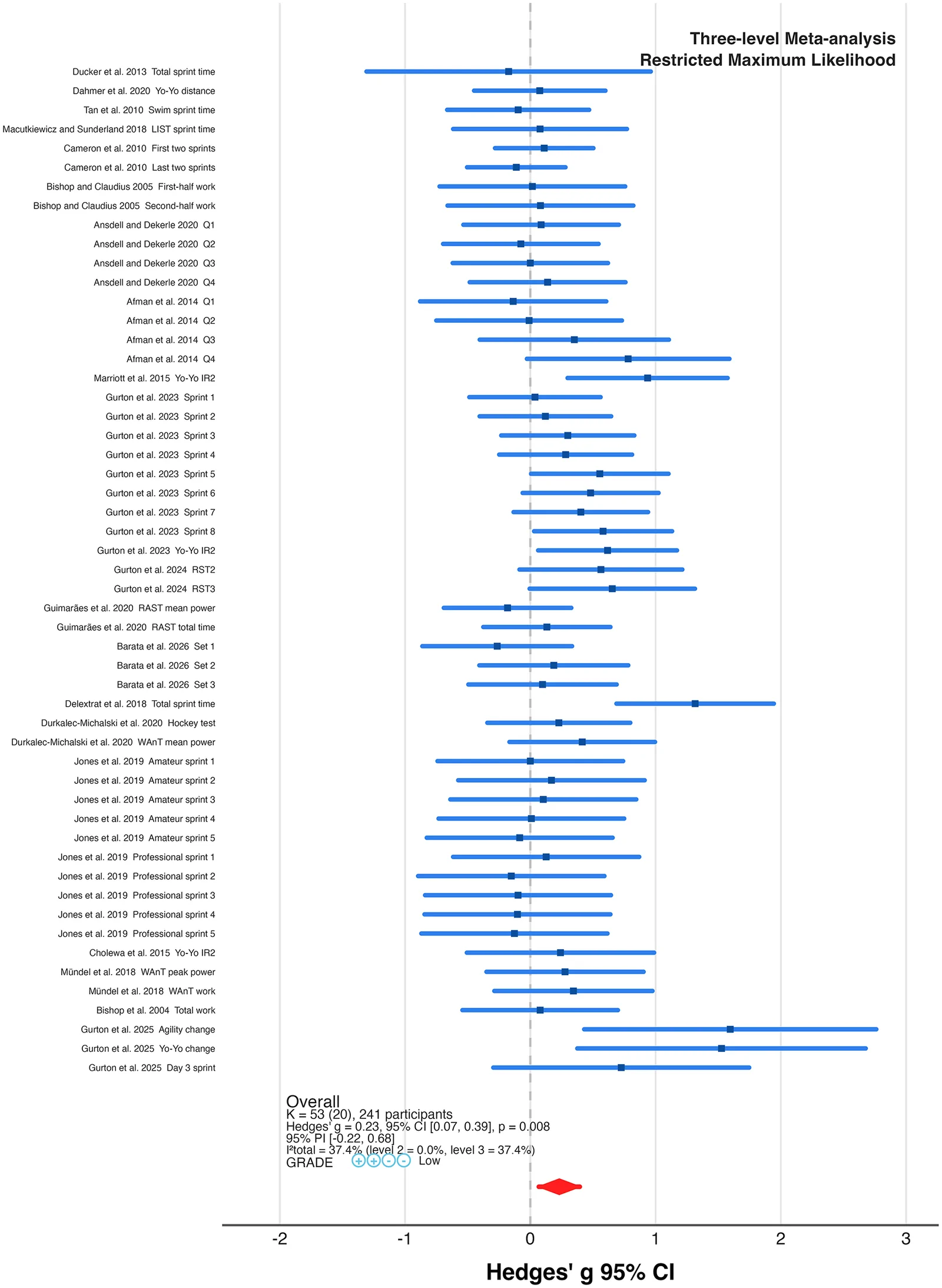
Effects of acute or short-term oral sodium bicarbonate supplementation on high-intensity intermittent exercise performance. Squares and horizontal lines represent individual Hedges' g values and 95% CIs; the red diamond represents the pooled three-level estimate. The dashed line indicates no effect, and positive values favor NaHCO_3_. K indicates effect estimates, with studies in parentheses. CI, confidence interval; GRADE, Grading of Recommendations Assessment, Development and Evaluation; LIST, Loughborough Intermittent Shuttle Test; PI, prediction interval; Q, quarter; RAST, Running-based Anaerobic Sprint Test; RST, repeated-sprint test; WAnT, Wingate Anaerobic Test; Yo-Yo IR2, Yo-Yo Intermittent Recovery Test Level 2.

The primary analysis included 53 performance effect sizes from 20 studies. The three-level random-effects meta-analysis showed a small beneficial effect of NaHCO_3_ on HIIE performance (Hedges' g = 0.23, 95% CI, 0.07 to 0.39; 95% PI, −0.22 to 0.68; df = 17.03; *P* = 0.008; Figure 3). The within-study heterogeneity variance was approximately zero, the between-study variance was 0.040, and total I^2^ was 37.36%. The Q test was not significant (Q (52) = 57.11, *P* = 0.291). When the analysis was restricted to trained team-sport athletes, the pooled effect remained similar (15 studies, 35 effect sizes; g = 0.26, 95% CI, 0.05 to 0.47; 95% PI, −0.30 to 0.82; df = 12.82; *P* = 0.018; I^2^ = 48.55%). Restriction to team-sport-specific HIIE protocols produced a comparable result (12 studies, 28 effect sizes; g = 0.24, 95% CI, 0.004 to 0.47; 95% PI, −0.31 to 0.78; df = 9.76; *P* = 0.047; I^2^ = 45.71%). Effect directions and magnitudes were consistent with the primary analysis, but both prediction intervals crossed zero and warrant cautious interpretation.

#### 3.5.2 Performance-domain analyses

**Figure 4:**
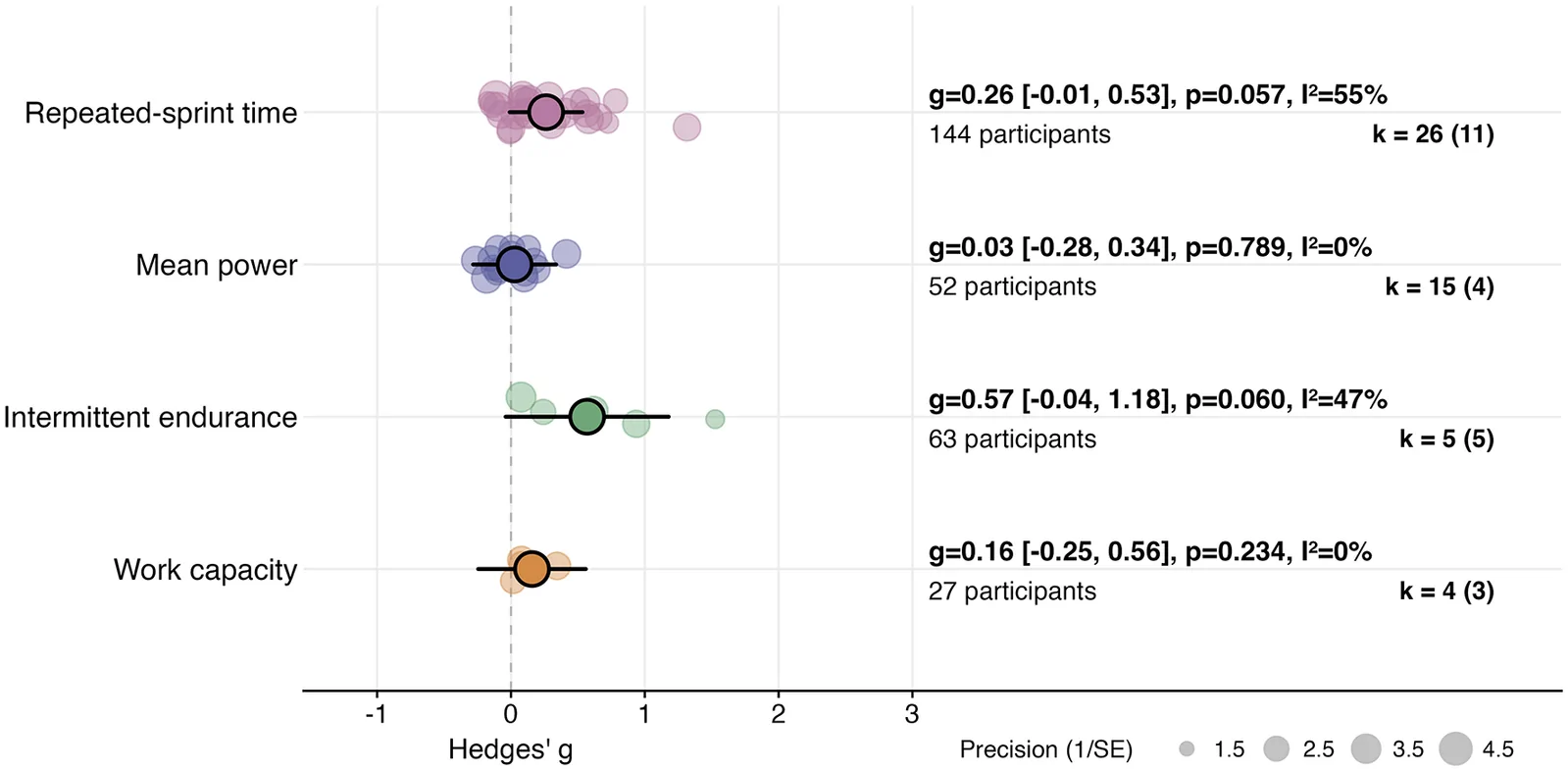
Orchard plot of exercise performance by domain. Small circles represent individual effects and are scaled by precision (1/SE); large circles and horizontal lines represent pooled Hedges' g and 95% CIs. Positive values favor sodium bicarbonate. I^2^, total heterogeneity; k, number of effect sizes, with studies in parentheses.

Prespecified domain analyses showed that the 95% CIs and 95% prediction intervals crossed zero for repeated-sprint time (11 studies, 26 effect sizes; g = 0.26, 95% CI, −0.01 to 0.53; 95% PI, −0.40 to 0.93; df = 8.86; *P* = 0.057; I^2^ = 55.12%), mean power (4 studies, 15 effect sizes; g = 0.03, 95% CI, −0.28 to 0.34; 95% PI, −0.28 to 0.34; df = 2.78; *P* = 0.789; I^2^ = 0%), intermittent endurance (5 studies, 5 effect sizes; g = 0.57, 95% CI, −0.04 to 1.18; 95% PI, −0.54 to 1.68; df = 3.64; *P* = 0.060; I^2^ = 47.38%), and work capacity (3 studies, 4 effect sizes; g = 0.16, 95% CI, −0.25 to 0.56; 95% PI, −0.25 to 0.56; df = 2.00; *P* = 0.234; I^2^ = 0%; Figure 4). Evidence was therefore insufficient to establish a clear effect within any specific performance domain. These findings should be interpreted cautiously because some domains included few studies.

#### 3.5.3 Physiological responses

Two-level meta-analyses showed that NaHCO_3_ increased post-exercise blood lactate (10 studies; MD = 1.73 mmol/L, 95% CI, 0.97 to 2.49; 95% PI, −0.08 to 3.54; df = 10; *P* < 0.001; I^2^ = 53.43%), blood ${\text{HCO}}_{3}^{-}$ (6 studies; MD = 2.73 mmol/L, 95% CI, 2.16 to 3.30; 95% PI, 2.16 to 3.30; df = 5; *P* < 0.001; I^2^ = 0%), and pH (7 studies; MD = 0.050, 95% CI, 0.025 to 0.075; 95% PI, −0.013 to 0.113; df = 6; *P* = 0.003; I^2^ = 70.27%). Detailed forest plots are provided in Supplementary Figures S1–S3.

#### 3.5.4 Subgroup analyses

**Figure 5:**
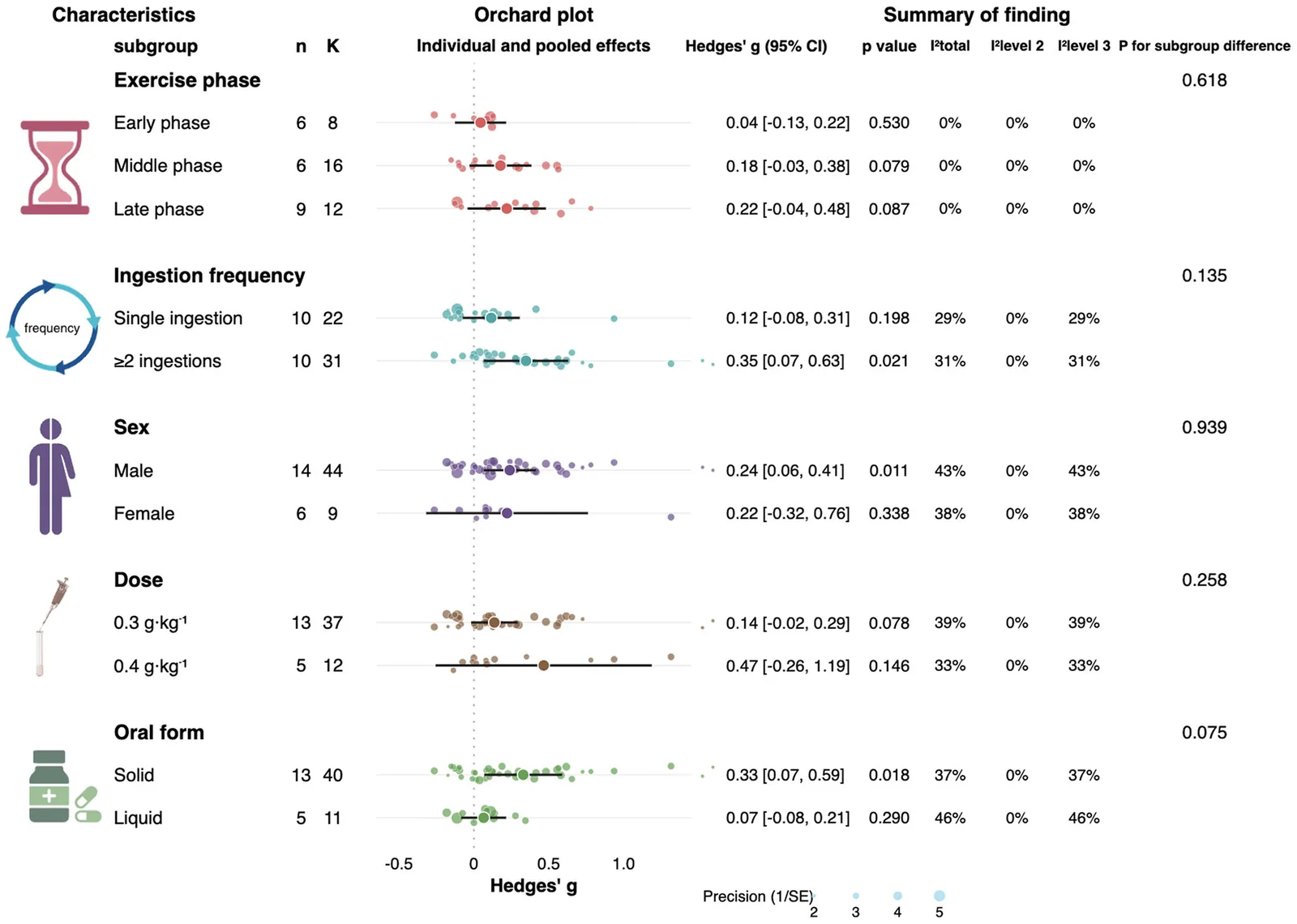
Subgroup analyses of high-intensity intermittent exercise performance. Small and large circles represent individual and pooled effects, respectively; horizontal lines indicate 95% CIs. Circle size is proportional to precision (1/SE). n, studies; K, effect sizes.

Subgroup analyses of HIIE performance showed significant pooled effects within the male (*P* = 0.011), ≥2-ingestion (*P* = 0.021), and solid-formulation (*P* = 0.018) subgroups (Figure 5). However, subgroup differences were not significant for sex (*P* = 0.939), dose (*P* = 0.258), ingestion frequency (*P* = 0.135), formulation (*P* = 0.075), or exercise phase (*P* = 0.618). Evidence was therefore insufficient to support moderation by these factors. Because some subgroups included few studies or were imbalanced, these findings were considered exploratory.

#### 3.5.5 Meta-regression

**Figure 6:**
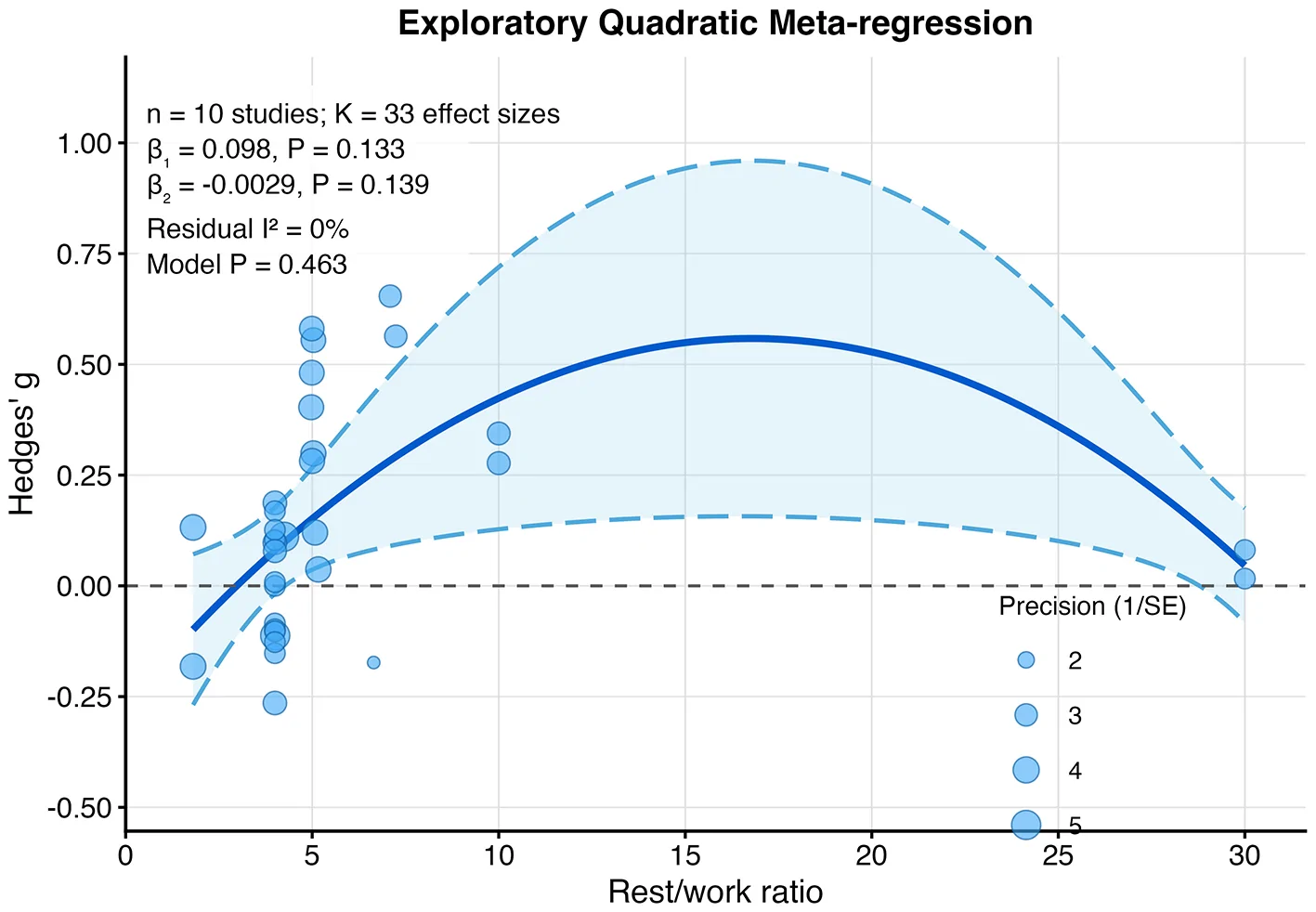
Exploratory quadratic meta-regression of the rest/work ratio and exercise performance. Circle size is proportional to precision (1/SE). The solid curve and shaded area represent the fitted model and 95% CI, respectively. n, studies; K, effect sizes.

The exploratory meta-regression of HIIE performance included 33 effect sizes from 10 studies. The rest/work ratio was not linearly associated with the supplementation effect (β = 0.004, *P* = 0.858). The quadratic term was also non-significant (β_2_ = −0.0029, *P* = 0.139; overall model *P* = 0.463), with low residual heterogeneity (residual I^2^ = 0%). Evidence was therefore insufficient to support either a linear or non-linear association. Given the limited number of studies and the small adjusted degrees of freedom, the non-significant findings may reflect low statistical power. The quadratic model was considered hypothesis-generating (Figure 6).

#### 3.5.6 Publication bias

**Figure 7:**
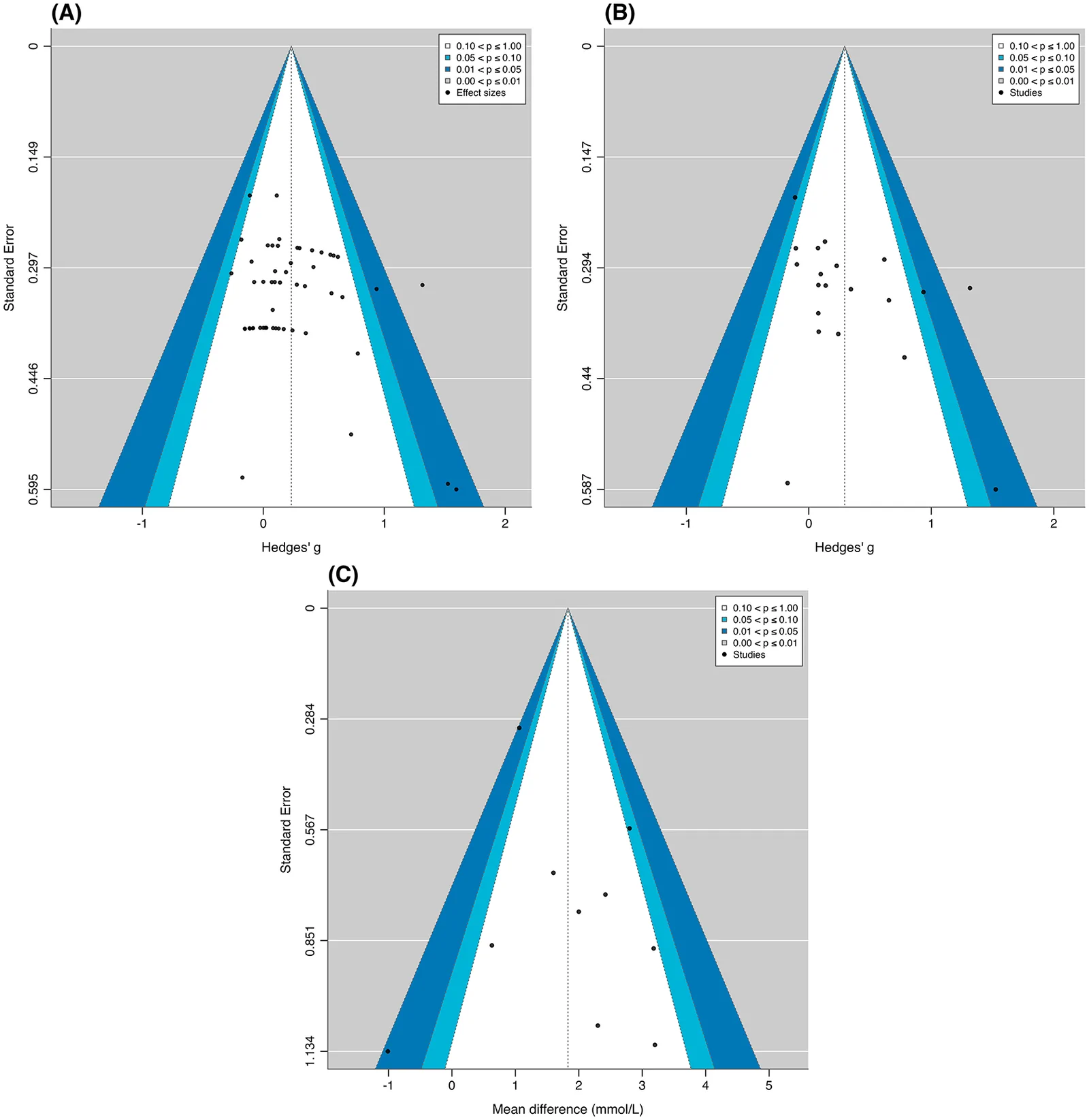
Contour-enhanced funnel plots for high-intensity intermittent exercise performance and blood lactate. Exercise performance was assessed at the effect-size level **(A)** and study level **(B)**, and blood lactate at the study level **(C)**. Shaded regions indicate two-sided significance contours, and dotted lines denote pooled effects.

For HIIE performance, the effect-size-level three-level Egger test did not indicate significant small-study effects (20 studies, 53 effect sizes; t = 1.62, *P* = 0.173). After retaining one prespecified primary effect per study, the study-level Egger test was also non-significant (z = 1.88, *P* = 0.077). The study-level Egger test for blood lactate was non-significant (10 studies; z = 0.07, *P* = 0.948). Given the limited number of studies, these findings do not exclude publication bias (Figure 7).

#### 3.5.7 Sensitivity analyses

**Figure 8:**
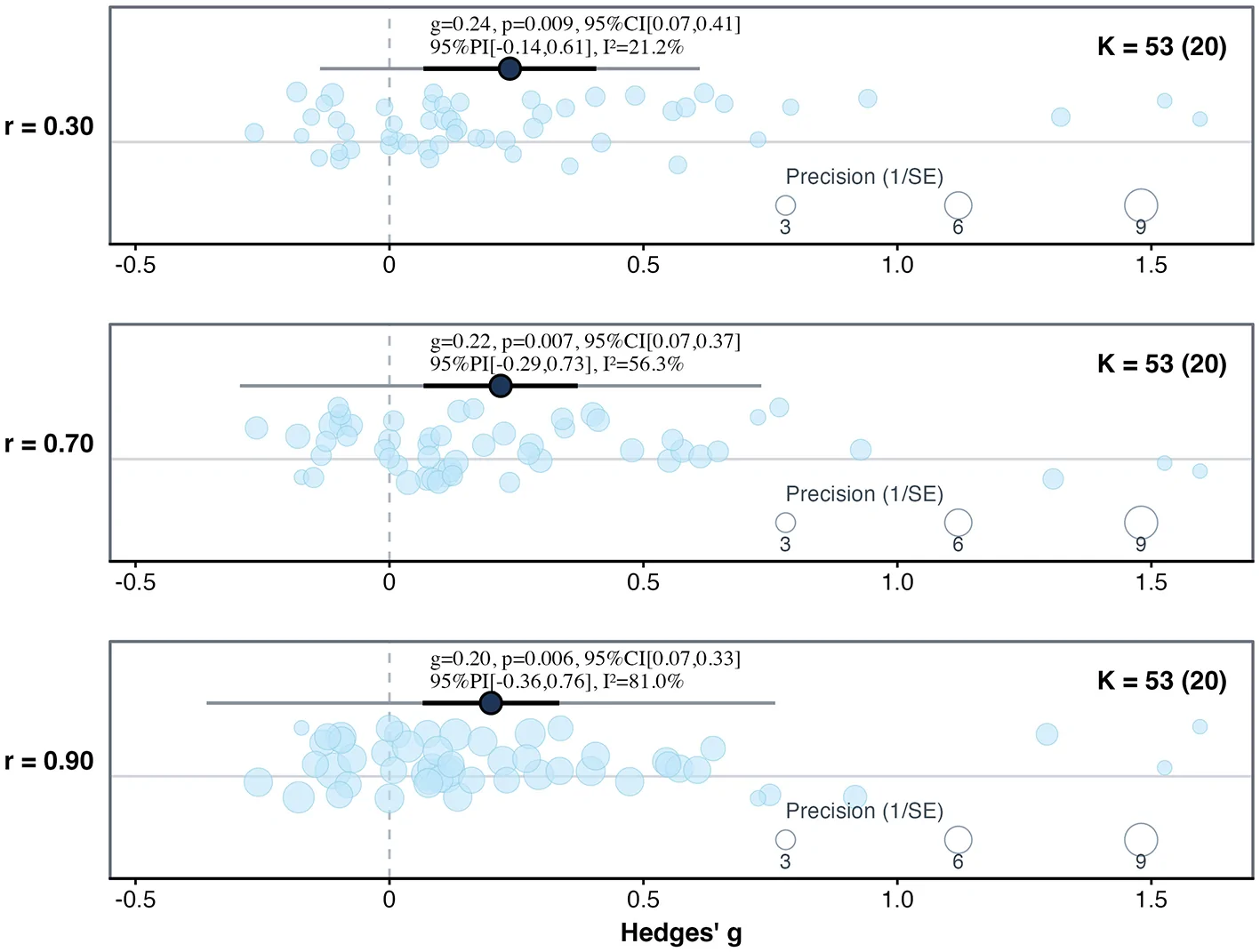
Sensitivity analysis using alternative paired correlations. Note. Panels show results for r = 0.30, 0.70, and 0.90. Bubble size represents precision (1/SE). Dark-blue circles and black and gray lines indicate pooled Hedges' g, 95% CIs, and 95% prediction intervals, respectively. K, effect sizes (studies).

Sensitivity analyses using alternative paired correlations showed stable pooled effects on HIIE performance across r values of 0.30, 0.70, and 0.90 (Hedges' g = 0.20–0.24, *P* = 0.006–0.009; Figure 8). Results were also consistent for blood lactate (MD = 1.70–1.76 mmol/L; all *P* < 0.001), ${\text{HCO}}_{3}^{-}$ (MD = 2.72–2.75 mmol/L; all *P* < 0.001), and pH (MD = 0.050–0.051; *P* = 0.002–0.003).

Sensitivity analyses of the assumed sampling correlation also produced stable effects on HIIE performance across ρ values of 0.30, 0.70, and 0.90 (Hedges' g = 0.21–0.24, *P* = 0.007–0.012). After retaining one prespecified primary effect per study, the pooled effect remained significant (Hedges' g = 0.29, 95% CI, 0.09 to 0.50; 95% PI, −0.30 to 0.89; df = 19; *P* = 0.007; I^2^ = 42.26%).

Leave-one-study-out analyses showed stable pooled effects on HIIE performance (Hedges' g = 0.17–0.25, *P* = 0.007–0.016). Effect directions and statistical conclusions were also unchanged for blood lactate (MD = 1.57–2.00 mmol/L, *P* < 0.001–0.002), ${\text{HCO}}_{3}^{-}$ (MD = 2.57–2.90 mmol/L; all *P* < 0.001), and pH (MD = 0.047–0.059, *P* < 0.001–0.010). Overall, the primary findings were not materially influenced by the correlation assumptions, outcome selection, or any single study (Supplementary Figures S4–S7).

#### 3.5.8 Gastrointestinal adverse events

Of the 20 included studies, 12 assessed or reported gastrointestinal symptoms, and 4 observed increases in some symptoms with NaHCO_3_. All 4 used 0.3 g·kg^−1^, but ingestion methods and timing differed. Cameron et al. (17) reported acute symptoms after a liquid formulation administered approximately 65 min before exercise, with greater flatulence and diarrhea during the subsequent 24 h. Guimarães et al. (43) observed higher stomach-pain and bloating scores after a solution ingested 90 min before exercise. Among capsule studies, Jones et al. (45) reported higher post-exercise gastrointestinal-symptom scores after ingestion approximately 90–100 min before exercise. Barata et al. (38) used divided doses, after which 3 participants reported belching, stomach pain, or abdominal cramping, whereas no corresponding symptoms occurred with PLA. Marriott et al. (49) reported gastrointestinal discomfort in 5 participants after 0.4 g·kg^−1^ in capsules, but did not establish whether symptoms were more frequent than with PLA or affected performance. The other 7 studies that assessed gastrointestinal tolerance found no greater symptom burden with NaHCO_3_ than with PLA. Macutkiewicz and Sunderland (46) reported scores of 0 for all symptoms, and Mündel et al. (48) reported no symptoms under either condition. Tan et al. (53), Delextrat et al. (47), and Gurton et al. (16, 39, 40) reported similar symptom prevalence or overall scores between conditions. A further 8 studies did not assess or adequately report gastrointestinal symptoms (10, 41, 42, 44, 50–52, 54). Overall, NaHCO_3_ may increase some gastrointestinal symptoms, but findings were inconsistent and adverse-event reporting remained incomplete.

## 4 Discussion

This systematic review evaluated acute or short-term oral NaHCO_3_ supplementation for team-sport-related HIIE performance and physiological responses. A total of 20 studies comprising 241 athletes and 53 performance effect sizes were included. The three-level model indicated a small beneficial effect on HIIE performance, although the 95% prediction interval crossed zero. Two-level models showed increases in post-exercise blood lactate, blood ${\text{HCO}}_{3}^{-}$, and pH. Subgroup analyses did not identify significant moderation by sex, dose, ingestion frequency, formulation, or exercise phase. Meta-regression found no significant linear or non-linear association between the rest/work ratio and the performance effect. However, the limited number of studies does not exclude potential moderation. Performance findings remained stable after varying correlation assumptions, retaining one primary effect per study, and sequentially excluding individual studies. Overall, NaHCO_3_ may provide a small average performance benefit and increase selected post-exercise physiological markers. However, prediction intervals crossed zero and certainty of evidence was low, suggesting that effects may vary across study conditions and individuals.

### 4.1 High-intensity intermittent exercise performance

Acute or short-term oral NaHCO_3_ supplementation produced a small beneficial effect on HIIE performance in team-sport athletes (Hedges' g = 0.23, *P* = 0.008). However, the 95% prediction interval (−0.22 to 0.68) crossed zero, indicating that true effects in different settings may range from no benefit to potential harm. Effect direction and statistical significance remained stable after varying paired and sampling correlations, retaining one primary effect per study, and conducting leave-one-study-out analyses. Thus, current evidence supports a small average benefit, but does not indicate that NaHCO_3_ consistently improves performance in all team-sport contexts.

Previous evidence on the ergogenic effects of NaHCO_3_ has been inconsistent and has largely focused on specific performance domains. A systematic review and meta-analysis by Lopes-Silva et al. (19) found no significant improvement in total work, best sprint, or final sprint performance during repeated-sprint exercise. A separate meta-analysis of the 30-s Wingate test also found no significant improvement in peak or mean power (55). Conversely, Grgic et al. (18) reported an approximately 16% improvement in exhaustive Yo-Yo intermittent-test performance. Individual studies have also reported benefits for Yo-Yo IR2 performance, intermittent-sprint work capacity, and repeated sprints after pre-fatigue (16, 49, 54). These discrepancies may reflect differences in metabolic demand and fatigue accumulation. Brief repeated sprints may be constrained more by the adenosine triphosphate-phosphocreatine (ATP-PCr) system, phosphocreatine resynthesis, and neuromuscular output (4). Longer intermittent tasks, more repetitions, or pre-fatigue may increase glycolytic involvement and acid-base disturbance, potentially providing more favorable conditions for extracellular buffering by NaHCO_3_ (9). Nevertheless, acid-base regulation is only one component of the complex fatigue process in team-sport HIIE and is unlikely to determine performance alone (4, 13). Improved acid-base status may therefore not consistently translate into better overall performance. Unlike reviews focused on a single exercise task, this study integrated multiple HIIE tasks and performance outcomes in team-sport athletes. A three-level model incorporating the within-study sampling variance-covariance structure was used to estimate the average effect across contexts.

To determine whether the overall effect was concentrated within a specific domain, we separately analyzed repeated-sprint time, mean power, intermittent endurance, and work capacity. No domain showed a clear effect of NaHCO_3_. Intermittent endurance had a moderate favorable point estimate (Hedges' g = 0.57, *P* = 0.060), but both the 95% confidence and prediction intervals crossed zero. This analysis included only 5 studies and had low adjusted degrees of freedom (df = 3.64), leaving considerable uncertainty. These results should not be interpreted as evidence that NaHCO_3_ is ineffective within these domains. Rather, current evidence is insufficient to establish that its effects are concentrated within any specific performance domain.

Subgroup analyses did not identify clear moderators. Although pooled effects were significant within the male, ≥2-ingestion, and solid-formulation subgroups, none of the corresponding subgroup differences was significant. These non-significant findings may reflect the small and imbalanced evidence base, heterogeneous protocols, doses concentrated at 0.3–0.4 g·kg^−1^, and limited subgroup power. Meta-regression was also non-significant. A single rest/work ratio may not adequately capture the overall metabolic demand of an exercise task, which also depends on bout duration, number of repetitions, recovery mode, total work, and outcome type. Given the limited number of independent studies and small adjusted degrees of freedom, evidence for moderation remains insufficient. Future studies should use larger samples and standardized team-sport-specific tests, prespecify the primary performance domain, and report phase-specific performance. Such work could clarify whether NaHCO_3_ is more effective during tasks with greater fatigue accumulation or in later exercise phases.

### 4.2 Physiological responses

Acute or short-term oral NaHCO_3_ significantly increased post-exercise blood ${\text{HCO}}_{3}^{-}$ (MD = 2.73 mmol/L) and pH (MD = 0.050), indicating the expected physiological alkalosis. These findings are consistent with previous syntheses. Calvo et al. (56) found that NaHCO_3_ increased exercise-related blood ${\text{HCO}}_{3}^{-}$, pH, and lactate. De Oliveira et al. (57) similarly reported increased blood ${\text{HCO}}_{3}^{-}$ and a small beneficial effect of extracellular buffering supplements on exercise performance. Physiologically, increased extracellular ${\text{HCO}}_{3}^{-}$ and pH enlarge the transmembrane H^+^ gradient and facilitate the transport of H^+^ and lactate out of contracting muscle. These responses may attenuate changes in intramuscular acid-base status and help maintain glycolytic energy provision and contractile output (11, 12).

Physiological alkalosis, however, is not sufficient to improve performance. Blood ${\text{HCO}}_{3}^{-}$ responses were relatively consistent, whereas pH showed substantial heterogeneity and a prediction interval that crossed zero. Leave-one-study-out analysis reduced pH heterogeneity to 0% after excluding Cholewa et al. (50), identifying this study as the primary source of heterogeneity. Nevertheless, the direction and statistical significance of the pooled effect were unchanged. Importantly, some studies reported increased blood ${\text{HCO}}_{3}^{-}$ and pH without improved performance (17, 46). Acid-base homeostasis therefore represents only one component of fatigue during HIIE. Inorganic phosphate accumulation, K^+^ homeostasis, Ca^2+^ release, phosphocreatine recovery, glycogen availability, and neuromuscular function may also limit performance (4, 13).

NaHCO_3_ also significantly increased post-exercise blood lactate (MD = 1.73 mmol/L). This response may reflect enhanced transport of lactate and H^+^ from working muscle into the blood, increased glycolytic flux, or a greater amount of work completed (56, 58). Lactate itself is not the direct cause of exercise-induced acidosis and can serve as a substrate for inter-tissue energy transport and oxidation (59, 60). However, peripheral blood lactate reflects the net balance of production, efflux, oxidation, and clearance. It neither directly represents intramuscular pH nor independently predicts a performance benefit. Because blood lactate showed substantial heterogeneity and a prediction interval that crossed zero, it should be viewed as supportive evidence of an altered metabolic response. Future studies should concurrently assess blood and intramuscular acid-base markers, work completed, and performance under standardized exercise conditions. This approach may clarify when physiological alkalosis translates into a performance benefit.

### 4.3 Gastrointestinal tolerance and individual variability

Oral NaHCO_3_ may cause stomach pain, bloating, flatulence, diarrhea, and abdominal cramping, although findings were inconsistent. Among the 12 studies reporting gastrointestinal responses, some observed a greater symptom burden, whereas others found no consistent difference from PLA. Studies reporting increased symptoms all used 0.3 g·kg^−1^ and included both liquid and capsule formulations, suggesting that discomfort cannot be attributed to a particular formulation alone. However, this was also the most frequently studied dose, and the pattern may reflect the distribution of available studies rather than greater risk. Other studies using 0.3–0.5 g·kg^−1^ found no clear increase in symptoms (47, 48, 53). Individual tolerance, ingestion strategy, feeding status, and assessment timing may all affect gastrointestinal responses. Current evidence is insufficient to determine whether dose, formulation, or ingestion timing primarily explains between-study differences.

These findings require cautious interpretation. A total of 8 studies did not assess or adequately report gastrointestinal symptoms, potentially underestimating their occurrence. Differences in symptom scales, assessment times, and severity classifications also limited comparisons and prevented reliable estimation of overall incidence and severity. Gastrointestinal discomfort may constrain the practical use of NaHCO_3_ and reduce its potential performance benefit (12, 61), but this possibility has not been adequately evaluated. The extent to which gastrointestinal symptoms offset any ergogenic benefit therefore remains uncertain. In practice, athletes should consider individual tolerance and test the dose, formulation, and ingestion timing during training to minimize adverse effects on performance and adherence.

### 4.4 Practical implications

Acute or short-term oral NaHCO_3_ may provide a small average performance benefit in team-sport athletes, but effects vary across individuals and exercise contexts. Increases in blood ${\text{HCO}}_{3}^{-}$ and pH can confirm physiological alkalosis, and higher blood lactate can support an altered metabolic response. However, these biomarkers do not demonstrate improved performance. No clear moderation was identified by sex, dose, ingestion frequency, formulation, or exercise phase, and current evidence does not support one protocol for all athletes. Athletes should test supplementation during training or match simulation and evaluate both sport-specific performance and gastrointestinal tolerance. Dose, formulation, and timing should be individualized, and NaHCO_3_ should not be used for the first time during competition. It may be a useful strategy for athletes who show a reproducible performance benefit and good tolerance. Continued use based solely on blood biomarkers is not warranted when no performance benefit is observed or discomfort occurs. NaHCO_3_ should therefore be guided by sport-specific testing and individual response rather than considered universally ergogenic.

### 4.5 Limitations

This review has several limitations. First, sample sizes in the included studies were generally small, which may have reduced the precision of effect estimates and the power of exploratory analyses. Male participants were also overrepresented, limiting generalizability to female athletes. Second, studies covered different team sports and testing protocols, with variation in exercise duration, recovery structure, number of repetitions, sport-specific actions, and outcomes. The approximate sampling variance-covariance matrix and three-level model addressed dependence among within-study effects but could not eliminate this clinical and methodological heterogeneity. Third, doses were concentrated at 0.3–0.4 g·kg^−1^, and ingestion frequency and formulation were unevenly distributed, limiting the ability to detect moderation by supplementation protocol. Fourth, most crossover trials did not report paired correlations between conditions, and sampling correlations among effects from the same analytic sample were unavailable. Variances and covariances therefore relied on assumed values. Although sensitivity analyses did not change the main conclusions, these assumptions may have affected precision. Most studies also lacked an accessible prospective analysis plan, which may further reduce certainty because of selective-reporting concerns. Fifth, few studies reported physiological outcomes, blood lactate and pH showed substantial heterogeneity, and their 95% prediction intervals crossed zero. These findings should therefore be treated as supportive evidence. Sixth, neither effect-size-level nor study-level Egger tests indicated significant small-study effects, but the limited number of independent studies reduced power and publication bias cannot be excluded. Finally, gastrointestinal adverse events could not be quantitatively synthesized because few studies reported them and symptom definitions, assessment tools, and measurement times differed. We could not determine whether gastrointestinal discomfort attenuated or offset the potential performance benefit of NaHCO_3_.

### 4.6 Future directions

Future research should move beyond confirming the physiological effects of NaHCO_3_ and determine when these responses translate into performance benefits. Studies should distinguish between types of team-sport-related tests and standardize reporting of protocol characteristics and phase-specific performance. This would help determine whether effects depend on test structure or fatigue accumulation. Blood ${\text{HCO}}_{3}^{-}$, pH, and lactate should be measured at key time points before, during, and after exercise, and gastrointestinal symptoms should be assessed using standardized tools. Such measurements could distinguish inadequate alkalosis, physiological responses without performance benefits, and benefits attenuated by gastrointestinal discomfort. Crossover trials should fully report paired data, treatment sequence, washout, period effects, and carryover. Repeated testing should also assess the stability of individual responses. Primary studies should calculate sample sizes *a priori* using an anticipated minimum important effect and improve representation of female athletes and different competitive levels. These improvements would clarify the athletes, protocols, and exercise contexts most likely to benefit from NaHCO_3_.

## 5 Conclusion

This systematic review and meta-analysis indicates that acute or short-term oral NaHCO_3_ supplementation may slightly improve HIIE performance in team-sport athletes. However, the 95% prediction interval crossed zero, suggesting that effects vary across individuals and exercise contexts. Sex, dose, ingestion frequency, formulation, exercise phase, and rest/work ratio showed no clear moderating effects. NaHCO_3_ increased post-exercise blood ${\text{HCO}}_{3}^{-}$, pH, and lactate, but these physiological responses did not necessarily translate into improved performance. Given the low certainty of evidence and individual variation in gastrointestinal tolerance, NaHCO_3_ should not be regarded as universally ergogenic. Its use should be guided by sport-specific performance testing and individual tolerance.

## Data Availability

The original contributions presented in the study are included in the article/Supplementary material, further inquiries can be directed to the corresponding author.
